# Shared Principles of Ethics for Infant and Young Child Nutrition in the Developing World

**DOI:** 10.1186/1471-2458-10-321

**Published:** 2010-06-08

**Authors:** Jerome Amir Singh, Abdallah S Daar, Peter A Singer

**Affiliations:** 1 McLaughlin-Rotman Centre for Global Health, University Health Network and University of Toronto, Toronto, Ontario, Canada; 2 Centre for the AIDS Programme of Research in South Africa, Durban, South Africa; 3 Joint Centre for Bioethics and Dalla Lana School of Public Health, University of Toronto, Toronto, Ontario, Canada

## Abstract

**Background:**

The defining event in the area of infant feeding is the aggressive marketing of infant formula in the developing world by transnational companies in the 1970s. This practice shattered the trust of the global health community in the private sector, culminated in a global boycott of Nestle products and has extended to distrust of all commercial efforts to improve infant and young child nutrition. The lack of trust is a key barrier along the critical path to optimal infant and young child nutrition in the developing world.

**Discussion:**

To begin to bridge this gap in trust, we developed a set of shared principles based on the following ideals: *Integrity; Solidarity; Justice; Equality; Partnership, cooperation, coordination, and communication; Responsible Activity; Sustainability; Transparency; Private enterprise and scale-up; and Fair trading and consumer choice*. We hope these principles can serve as a platform on which various parties in the in the infant and young child nutrition arena, can begin a process of authentic trust-building that will ultimately result in coordinated efforts amongst parties.

**Summary:**

A set of shared principles of ethics for infant and young child nutrition in the developing world could catalyze the scale-up of low cost, high quality, complementary foods for infants and young children, and eventually contribute to the eradication of infant and child malnutrition in the developing world.

## Background

Despite potentially very large demand for high-quality, low-cost commercially produced fortified complementary foods (i.e., food given in addition to breast milk) for infants aged 6-24 months, the commercial sector has been slow to develop such products for the developing world [1]. If industry rises to this challenge by producing affordable, nutritious products and creating demand through appropriate marketing campaigns, such an approach could result in improved feeding and nutritional status and a corresponding decrease in death and disability in the developing world. The lack of private sector participation in this area is closely linked to the issue of trust, the lack of which is a key barrier along the critical path to optimal infant and young child nutrition in the developing world.

In June 2008, the Pacific Health Summit was held in Seattle, Washington. The topic focussed on global nutrition challenges. Delegates comprised approximately 150 individuals from a range of nutrition-related constituencies. We noted that there was consensus amongst delegates that the erosion of trust among various stakeholders - for example, breastmilk advocates, international bodies such as UNICEF, infant nutritionists, pediatricians, governments, and industry - in the infant feeding arena was undermining the scale-up of complementary feeding products for infants between the ages of 6-24 months in the developing world during the critical transition period from exclusive breastfeeding to total reliance on family foods. Inspired by shared principles of ethics that have been drafted to bring diverse groups together on common concerns and issues [2-4], we proposed to draft a set of shared ethics principles on complementary feeding as a confidence-building attempt to seed trust amongst stakeholders involved in the infant feeding arena.

*The Shared Principles of Ethics for Infant and Young Child Nutrition in the Developing World *described herein is intended to unite stakeholders in a shared vision and common goal: reduce and eventually eliminate childhood malnutrition in the developing world through the scale-up of low cost, high quality complementary foods, using home available or locally procured ingredients wherever feasible. The principles were drafted after a review of sources that have relevance to virtually all stakeholders and constituencies in the infant feeding arena. These include civil society codes of conduct, corporate social responsibility frameworks, governance frameworks, global health ethics frameworks, international health guidelines, consumer rights frameworks, and human rights instruments. The proposed principles are intended to complement and supplement existing laws, trade regimes, international Codes, and international goals (see Additional file 1). In this paper, we outline our proposed shared principles for stakeholders in the complementary feeding arena.

## Discussion

### Benefits of complementary feeding for infants aged 6-24 months

The World Health Organization recommends exclusive breastfeeding for the first 6 months of life, and continued breastfeeding until two years of age. However, after 6 months, breast milk alone is inadequate to meet dietary needs, both in terms of calories and vitamins and mineral of growing children. Therefore, WHO also recommends after 6 months the introduction of nutrient rich complementary foods [5].

Optimal breastfeeding could save about 1.5 million lives per year, almost all of which would be in the developing world. Evidence also indicates that micronutrient-enriched complementary feeding amongst infants aged 6-24 months play an important role in countering micronutrient deficiencies and could play an important role in lowering childhood mortality [6-9]. The Copenhagen Consensus is a process whereby economists conduct cost-effectiveness analyses of various interventions to tackle global problems (and not just in health). For the 2008 Copenhagen consensus - which focused on infant and child nutrition - over 50 economists worked for two years, and presented their arguments to a panel of eight top economists, including 5 Nobel Laureates, who assessed the research and made the final selections. After considering more than 40 proposed interventions to counter infant and child malnutrition, the panel proposed its top solutions with estimates of costs and benefits. The top two were: (1) Micronutrient Supplementation, involving the provision of Vitamin A capsules and therapeutic zinc supplements for under two-year-olds[10]. This would cost a total of $60.4 million annually, with benefits worth more than $1 bn yearly (benefit cost ration [BCR] of more than 17:1); and (2) Micronutrient Fortification, which would entail the provision of iodized salt and iron; This would cost $286 million annually, while the corresponding benefits are $2.7 bn (BCR of 9.5:1)[11]. Although the provision of micronutrient supplements was ranked highest amongst proposed interventions, the panel recognised that its provision, alone, wouldn't suffice to counter infant and child nutrition. Rather, it would ideally be part of a package of neonatal care, which would include de-worming, nutrition screening, and measles vaccination. The provision of affordable, commercially produced complementary foods could also provide needed micronutrients but would necessitate buy-in from all stakeholders involved in the infant and child feeding arena. This may be challenging given the erosion of trust in this sector.

### The erosion of trust in the infant feeding arena

Trust entails "a firm belief or confidence in the honesty, integrity, reliability, justice" as a condition of some relationship [12]. The defining event in the area of infant feeding is the aggressive marketing of infant formula in the developing world by transnational companies in the 1970s. This practice shattered the trust of the global health community in the private sector and culminated in a global boycott of Nestle products. This boycott persists today in at least 18 countries [13].

In response to such marketing practices, the World Health Organization drafted the *International Code on the Marketing of Breastmilk Substitutes *[14] in 1981, The Code recommends, amongst other measures, restrictions on the marketing of breastmilk substitutes, such as infant formula, to ensure that mothers are not discouraged from breastfeeding and that substitutes are used safely, if needed., However, evidence that some transnational companies have continued to promote substitute feeding products over breast-milk [15-21], in violation of the International Code, has further stigmatized the private sector in this arena. The resulting anti-industry sentiment in some quarters has unfortunately distracted attention from the potential value [6-9] of complementary feeding products for infants aged 6-24 months. Moreover, past transgressions of the Code on the part of industry related to both formula promotion has have fuelled suspicions that industry will exploit the opportunity to promote complementary products to push supplementary products for infants aged under 6 months of age. This is true for companies who have been in violation, and even those who have not. There has been a "chilling effect" on companies getting into the complementary food for 6-24 month space. Industry, in turn, has become suspicious of breast-milk advocates and activists, seeing such parties as hostile to their business interests and unnecessarily wary of their humanitarian goals.

Consequently, industry has been reluctant to play a meaningful role in scaling-up complementary products in markets where there is relatively small profits, for fear of harming their global brand. This, in turn, may be reducing human capital potential given the important role of adequate nutrition in the 6-24 month period for growth and development.

To begin to bridge this gap in trust, we developed a set of shared principles as a starting point (see Additional File 2 for the proposed principles, without the accompanying commentary).

## Shared principles - and commentaries

### Principle one Integrity

In pursuit of our common goal, we sincerely commit to basing our actions on the values enunciated in this document.

We agree to set specific goals together, and to monitor and report results.

We reject corruption and activities that undermine good governance.

#### Commentary

Integrity is central to trust. However, corrupt practices can undermine trust and threaten important initiatives. Governments have recognized this factor and several regional political bodies have promulgated treaties outlawing corruption [22-24]. At the wider international level, the United Nations General Assembly has passed a resolution condemning corruption [25], while the United Nations Convention against Corruption [26] is the leading international anti-corruption instrument. In the context of complementary feeding of infants, the principle of integrity is intended to encourage all stakeholders to act with integrity and to refrain from engaging in questionable practices, such as promoting breastmilk substitute products which could undermine exclusive breastfeeding in an infant's first 6 months of life, and their continued breastfeeding with adequate complementary feeding from 6-24 months of age.

### Principle two Solidarity

We recognize that humans have a moral responsibility towards each other. We affirm our commitment to ensure the common welfare of humankind, particularly the poor and marginalised in developing countries. We pledge our commitment to relieving human suffering and saving lives in pursuit of our common goal.

In the context of infant and young child nutrition, the principle of solidarity is intended to encourage all stakeholders in the infant feeding area to act in the interest of malnourished infants everywhere by supporting initiatives that could alleviate their plight.

#### Commentary

The concept of solidarity relates to "the notion of cooperation, common rights and responsibilities as well as unity for the achievement of a common goal" [27]. At a national level, solidarity has found expression in many countries in various forms of welfare benefits. At the international level, solidarity is well recognised within the United Nations system, as epitomized by the establishment of international humanitarian agencies such as UNICEF, and the offices of the High Commissioner for Refugees and High Commissioner for Human Rights (the latter of which created the specialised post of independent expert on human rights and international solidarity) [28]. The concept of solidarity is also explicitly recognised in the United General Assembly as evidenced by its passage of resolutions such as International Solidarity with the Liberation Struggle in South Africa during the apartheid era [29] and the declaration of a dedicated International Human Solidarity Day [30] as an initiative in the fight against poverty. The principle of solidarity is also a key pillar of the global health ethics paradigm [31,32]. In the context of complementary feeding, the principle of solidarity is intended to encourage all stakeholders in the infant feeding area to act in the interest of malnourished infants everywhere by supporting initiatives that could alleviate their plight.

### Principle three Justice

We recognize the importance of eradicating health inequality between different population groups. We will, at all times, adopt the principle of fairness in each program and activity being implemented in furtherance of attaining our common goal.

To achieve our common goal, and cognizant of extreme levels of poverty in some settings, we recognize the need to keep the costs of complementary foods for infants and children low to facilitate its access by those in need.

#### Commentary

The notion of justice is central to law and human rights. Similarly, the principle of justice is one of the four pillars of the biomedical principles of ethics [33], which are arguably the most cited guiding principles in the field of bioethics. In the context of health, the notion of justice is framed as a consideration of fairness and distribution. In the context of complementary feeding, the principle of justice is intended to remind stakeholders that they have a moral duty to facilitate access to low cost, high quality complementary foods for infants aged 6-24 months.

### Principle four Equality

In pursuit of our common goal, we undertake not to unfairly discriminate on the basis of nationality, wealth, age, gender, race, ethnicity, color, social background, social status, sexual orientation, and class.

In the context of infant and young child nutrition, despite women overwhelmingly being the primary caregivers everywhere, most policies are drafted without their involvement or input. This principle is intended to draw attention to this practice and to encourage stakeholders to prospectively consult women when policy formulation related to complementary infant nutrition occurs.

Accordingly, we pledge, at all times, to consult women in making decisions and formulating policies, as well as in seizing opportunities, in relation to achieving our common goal.

#### Commentary

The principle of equality has its roots in the notion of egalitarianism. Egalitarianism asserts that all people are of equal value and should be treated the same irrespective of their origin or personal characteristics. In modern times, the notion of equality is explicitly outlined in the Universal Declaration of Human Rights. Its primacy in human rights is underscored by its expression as the first right enumerated in the Declaration.

### Principle five Partnership, cooperation, coordination, and communication

We undertake to foster partnerships, relationships and cooperation with all stakeholders in good faith - including governments, private sector, donor agencies, international institutions, consumers, and civil society - in efforts to accomplish our common goal.

We undertake to communicate actively with each other. Such collaboration shall be underpinned by meaningful and good faith dialogue, and abide by the principles of equality, openness, partnership, mutual respect, authentic trust, and professionalism.

We undertake to create a forum to coordinate and facilitate such interaction with each other.

#### Commentary

Several cooperation, coordination, and communication governance instruments and bodies exist at the international level, perhaps best highlighted by the Organisation for Economic Co-operation and Development (OECD). The inclusion of a principle on partnership, cooperation, coordination, and communication is intended to remind stakeholders in the infant feeding arena that they can only achieve the undisputed goal of reducing, and eventually eliminating, childhood nutrition if they work together on tackling the matter. It is also intended to emphasize that mutual respect and communication is central to establishing and maintaining trust.

### Principle six Responsible activity

In pursuit of our common goal, we commit ourselves to activities that comply with international codes and where applicable, domestic laws.

We commit ourselves to responsible activity and evidence-based decisions.

In the context of infant and young child nutrition, we commit to following the International Code of Marketing of Breastmilk Substitutes.

#### Commentary

The disregard of the International Code of Marketing of Breastmilk Substitutes by some industry players, and the disregard of evidence-based science for political reasons in some settings [35] highlight the need to include a guiding principle on responsible activity. This principle is intended to affirm respect for the rule of law and other relevant governance instruments.

### Principle seven Sustainability

We commit to ensuring sustainable utilization of natural resources and the environment in pursuit of our common goal.

We commit ourselves to cost-effective complementary foods.

We commit ourselves to environmentally-friendly production, packaging, and distribution of complementary foods.

We recognize the important role of local labor in achieving our common goal.

#### Commentary

The sustainable use of natural resources is key to human survival. This is epitomized in the convening of 1992 Earth Summit in Rio de Janeiro and the 2002 World Summit on Sustainable Development in Johannesburg. In the context of infant nutrition, sustainability entails ensuring that complementary feeding products originate from sustainable sources. Moreover, that local labor should play a key role in the scale-up of complementary foods and related products, where possible.

### Principle eight Transparency

We undertake to conduct our activities towards our common goal in an open and transparent manner.

#### Commentary

Governments ought to engage in transparent decision-making as it promotes accountability, acts as a check against corruption and mismanagement, and instills public confidence. Similarly, Transparency Declarations are key to corporate governance in many jurisdictions. At an international level, transparency is the central theme of the 1993 UNCITRAL Model Law on Procurement of Goods, Construction and Services [36] and in the UN International Code of Conduct for Public Officials, published as an annex to the United Nations General Assembly resolution on corruption [37]. In the context of infant and young child nutrition, the inclusion of a principle on transparency is intended to encourage stakeholders in the infant feeding arena to conduct their affairs in relation to complementary feeding in a transparent manner so as to facilitate the building of trust and confidence.

### Principle nine Private enterprise and scale-up

We acknowledge a potential role of private enterprise, including community entrepreneurs, in scaling-up production of low-cost, high quality complementary foods and related products for infants and young children in developing countries in achieving our common goal.

We recognise the need to explore innovative business models, in urban and rural areas, to achieve our common goal.

#### Commentary

As noted above, industry has unparalleled experience in scaling up the production of feeding products. Their input in scaling-up the production of complementary feeding products will be vital to a rollout campaign of such products and to sustainability. The inclusion of a principle on private enterprise and scale-up is intended to acknowledge its crucial role in this regard, and to encourage other stakeholders to support them in achieving this goal for the benefit of the undernourished.

### Principle 10 Fair trading and consumer choice

We recognize the importance of a fair market and fair commercial practices in achieving our common goal.

In pursuit of our common goal we recognize the consumer's right to receive appropriate information to enable him/her to make an informed choice on low-cost, high quality complementary foods and related products for infants and young children.

We recognize the need for accurate information and efficient, transparent and accountable regulatory systems in countries where the products are to be disseminated.

#### Commentary

Many countries have passed consumer protection laws to ensure fair competition and the free flow of truthful information in the marketplace. Such laws are intended to protect the interests of consumers. Similarly, this principle is intended to emphasize that consumers are key stakeholders in the complementary feeding arena, and that they have a right to truthful and relevant information to make informed decisions about complementary feeding products. This principle is also intended to emphasize that governments have a key role to play in ensuring that effective regulatory guidelines are established to promote and protect consumer rights.

## Summary

We recognise that no set of principles is a panacea that will automatically restore trust in a situation where it has been lost, such as in the infant and young child nutrition arena. However, the shared principles of ethics set forth herein can create an enabling environment for those who must work closely together to save lives, to begin to do so. There is a huge opportunity cost (i.e., the best alternative that is forgone because a particular course of action is pursued), measured in millions of lives, of not collaborating and engaging the private sector in scaling up of infant and young child nutrition. The shared principles of ethics outlined here are a modest attempt, but a start that might bring to the table people who normally rarely find themselves in the same room. We are also aware that our approach may very well not work if any of the parties does not genuinely want dialogue, believing that their current approach is serving them well. It is our hope that in making such principles explicit, the shared principles of ethics can serve as a platform on which various parties in the complementary feeding arena, and the broader arena of infant and young child nutrition, can begin a process of authentic trust-building that will ultimately result in coordinated efforts amongst parties. We also hope the proposed set of principles and their subsequent evolution will catalyze the scale-up of low cost, high quality, complementary foods for infants and young children, and eventually result in the eradication of infant and child malnutrition in the developing world.

## Authors' contributions

PAS conceptualized the idea of an ethical code for infant and young child nutrition. JAS developed the code and drafted the manuscript with input from ASD and PAS. All authors read and approved the final manuscript.

## Competing interests

The authors serve as advisors to the Bill & Melinda Gates Foundation through grants funded by the Grand Challenges in Global Health Initiative and the Water Efficient Maize for Africa (WEMA) initiative.

Drs. Daar and Singer have in the past received consulting funds from Pepsico Inc unrelated to this topic.

## Pre-publication history

The pre-publication history for this paper can be accessed here:

http://www.biomedcentral.com/1471-2458/10/321/prepub

## Supplementary Material

Additional file 1 **Existing laws, trade regimes, international codes, and international goals relevant to infant and child nutrition**. This file contains the existing primary international instruments and position statements relevant to infant and child nutrition. Click here for file

Additional file 2 **Existing laws, trade regimes, international codes, and international goals relevant to infant and child nutrition**. This file contains the existing primary international instruments and position statements relevant to infant and child nutrition. Click here for file
