# Supplementary material for: Shared Principles of Ethics for Infant and Young Child Nutrition in the Developing World
Source: BMC Public Health. 2010 Jun 8;10:321. doi: 10.1186/1471-2458-10-321 (PMC2906463; doi:10.1186/1471-2458-10-321)
Supplement: Additional file 1 — Existing laws, trade regimes, international codes, and international goals relevant to infant and child nutrition. This file contains the existing primary international instruments and position statements relevant to infant and child nutrition. [file 1471-2458-10-321-S1.DOC]

**Additional File 1**

| **Key international instruments governing, and relevant to, infant health and nutrition**  1. Constitution of the World Health Organisation. Adopted by the International Health Conference held in New York from 19 June to 22 July 1946, signed on 22 July 1946, and entered into force on 7 April 1948. [Particularly, the preamble (‘The enjoyment of the highest attainable standard of health is one of the fundamental rights of every human…’; and (‘Healthy development of the child is of basic importance’)].  2. The Universal Declaration of Human Rights. Proclaimed by the United Nations General Assembly in Paris on 10 December 1948 [General Assembly resolution 217 A (III)](http://daccess-ods.un.org/access.nsf/Get?Open&DS=A/RES/217(III)&Lang=E). 3. International Covenant on Economic, Social and Cultural Rights. Adopted and opened for signature, ratification and accession by General Assembly resolution 2200A (XXI) of 16 December 1966. Entry into force 3 January 1976. [Particularly article 12(1) (The States Parties to the present Covenant recognize the right of everyone to the enjoyment of the highest attainable standard of physical and mental health] and 12(2)(1) (The steps to be taken by the States Parties to the present Covenant to achieve the full realization of this right shall include those necessary for …the provision for the reduction of … infant mortality and for the healthy development of the child.)] 4. World Health Organization. International Code of Marketing of Breastmilk Substitutes. Geneva , World Health Organization; 1981.  5. World Health Assembly resolutions on infant and young-child nutrition, appropriate feeding practices and related questions (particularly WHA27.43, WHA31.47, WHA31.55, WHA32.42, WHA33.32, WHA34.22, WHA 32.42, WHA35.26, WHA37.30, WHA39.28, WHA41.11, WHA43.3, WHA44.33, WHA45.34, WHA46.7, WHA47.5, WHA49.15, WHA54.2, WHA 55.25, WHA58.32, and WHA59.21). 6. Convention on the Rights of the Child. Adopted and opened for signature, ratification and accession by General Assembly resolution 44/25 of 20 November 1989. Entry into force 2 September 1990. 7. World Declaration and Plan of Action on Nutrition, adopted by the International Conference on Nutrition of 1992. 8. FAO/WHO 1994 Food Standards Program Codex Alimentarious Commission. Annex F: Guidelines on Formulated Supplementary Foods for Older Infants and Young Children. 9. United Nations Millennium Development Goals of 2000 [Goal 4: Reduce Child Mortality]. |
| --- |
