# Supplementary material for: Shared Principles of Ethics for Infant and Young Child Nutrition in the Developing World
Source: BMC Public Health. 2010 Jun 8;10:321. doi: 10.1186/1471-2458-10-321 (PMC2906463; doi:10.1186/1471-2458-10-321)
Supplement: Additional file 2 — Existing laws, trade regimes, international codes, and international goals relevant to infant and child nutrition. This file contains the existing primary international instruments and position statements relevant to infant and child nutrition. [file 1471-2458-10-321-S2.DOC]

**Additional File 2**

| **Shared principles of ethics on complementary feeding products for infants and children in the developing world**  The following principles outline our common commitment to reducing, and eventually eradicating, childhood malnutrition. They are intended to engender trust amongst signatories.  These principles complement and supplement existing laws, trade regimes, international Codes, and international goals. Adoption of these principles is voluntary and draws on a range of existing codes, norms, standards and guidelines.  The signatories (hereafter “we” and “our”) to these shared principles agree to apply the principles to all our policies, activities and operations in relation to our common goal of reducing and eventually eliminating childhood malnutrition in the developing world through the scale-up of low cost, high quality complementary feeding products (hereinafter “common goal”).  These principles do not replace existing codes or practices to which signatories may also be party, except as specified by them. Their adoption does not prevent signatories from supporting or using other tools to promote transparency and accountability. We undertake to adhere to the International Code of Marketing of Breast-milk Substitutes (1981; [Resolution WHA34.22](http://www.babymilkaction.org/regs/res3422.html)).  We will refine these principles through experience, taking into account future developments, particularly those that improve accountability and transparency.  **Preamble**  Acknowledging that childhood malnutrition is one of the world’s most urgent concerns given that malnutrition impedes growth and cognitive development in children, which in turn reduces their ability to learn and diminishes their productivity in adulthood, we hereby commit ourselves to achieving our common goal.  We undertake this commitment in good faith, acknowledging that stakeholder cooperation offers the best chance of facilitating solutions to achieving our common goal.  We take earnestly our duties in respect of, amongst others, social responsibility, sustainable development, corporate citizenship, and civic responsibility, as the case may be.  ***Principle one***  ***Integrity***  In pursuit of our common goal, we sincerely commit to basing our actions on the values enunciated in this document.  We agree to set specific goals together, and to monitor and report results.  We reject corruption and activities that undermine good governance.  ***Principle two***  ***Solidarity***  We recognize that humans have a moral responsibility towards each other. We affirm our commitment to ensure the common welfare of humankind, particularly the poor and marginalised in developing countries. We pledge our commitment to relieving human suffering and saving lives in pursuit of our common goal.  In the context of infant and young child nutrition, the principle of solidarity is intended to encourage all stakeholders in the infant feeding area to act in the interest of malnourished infants everywhere by supporting initiatives that could alleviate their plight.  ***Principle three***  ***Justice***  We recognize the importance of eradicating health inequality between different population groups. We will, at all times, adopt the principle of fairness in each program and activity being implemented in furtherance of attaining our common goal.  To achieve our common goal, and cognizant of extreme levels of poverty in some settings, we recognize the need to keep the costs of complementary foods for infants and children low to facilitate its access by those in need.  ***Principle four***  ***Equality***  In pursuit of our common goal, we undertake not to unfairly discriminate on the basis of nationality, wealth, age, gender, race, ethnicity, color, social background, social status, sexual orientation, and class.  In the context of infant and young child nutrition, despite women overwhelmingly being the primary caregivers everywhere, most policies are drafted without their involvement or input. This principle is intended to draw attention to this practice and to encourage stakeholders to prospectively consult women when policy formulation related to complementary infant nutrition occurs.  Accordingly, we pledge, at all times, to consult women in making decisions and formulating policies, as well as in seizing opportunities, in relation to achieving our common goal.  ***Principle five***  ***Partnership, cooperation, coordination, and communication***  We undertake to foster partnerships, relationships and cooperation with all stakeholders in good faith – including governments, private sector, donor agencies, international institutions, consumers, and civil society – in efforts to accomplish our common goal.  We undertake to communicate actively with each other. Such collaboration shall be underpinned by meaningful and good faith dialogue, and abide by the principles of equality, openness, partnership, mutual respect, authentic trust, and professionalism.  We undertake to create a forum to coordinate and facilitate such interaction with each other.  ***Principle six***  ***Responsible activity***  In pursuit of our common goal, we commit ourselves to activities that comply with international codes and where applicable, domestic laws.  We commit ourselves to responsible activity and evidence-based decisions.  In the context of infant and young child nutrition, we commit to following the International Code of Marketing of Breastmilk Substitutes.  ***Principle seven***  ***Sustainability***  We commit to ensuring sustainable utilization of natural resources and the environment in pursuit of our common goal.  We commit ourselves to cost-effective complementary foods.  We commit ourselves to environmentally-friendly production, packaging, and distribution of complementary foods.  We recognize the important role of local labor in achieving our common goal.  ***Principle eight***  ***Transparency***  We undertake to conduct our activities towards our common goal in an open and transparent manner.  ***Principle nine***  ***Private enterprise and scale-up***  We acknowledge a potential role of private enterprise, including community entrepreneurs, in scaling-up production of low-cost, high quality complementary foods and related products for infants and young children in developing countries in achieving our common goal.  We recognise the need to explore innovative business models, in urban and rural areas, to achieve our common goal.  ***Principle 10***  ***Fair trading and consumer choice***  We recognize the importance of a fair market and fair commercial practices in achieving our common goal.  In pursuit of our common goal we recognize the consumer’s right to receive appropriate information to enable him/her to make an informed choice on low-cost, high quality complementary foods and related products for infants and young children.  We recognize the need for accurate information and efficient, transparent and accountable regulatory systems in countries where the products are to be disseminated. |
| --- |
